# Elevated Urbanization-Driven Plant Accumulation of Metal(loid)s Including Arsenic Species and Assessment of the Kłodnica River Sediment Contamination

**DOI:** 10.1007/s00244-022-00967-y

**Published:** 2022-11-16

**Authors:** Magdalena Jabłońska-Czapla, Katarzyna Grygoyć

**Affiliations:** Institute of Environmental Engineering of the Polish Academy of Sciences, 34 M. Skłodowska-Curie Street, 41-819 Zabrze, Poland

## Abstract

The impact of water and bottom sediment pollution of a river subjected to a strong industrial anthropogenic pressure of metal(loid) (including arsenic and its species) accumulation in riverbank plants such as *Solidago virgaurea* L., *Phragmites* L. and *Urtica dioica* L. was investigated. The high-performance liquid chromatography-inductively coupled plasma-mass spectrometry (HPLC-ICP-MS) technique was used to study organic and inorganic arsenic species in selected plants and their response to heavy metal and arsenic contamination. The modified BCR extraction results showed that arsenic was mainly bound to the mobile reducible and organic-sulfide fractions in the Kłodnica River bottom sediments. Research has shown that the bottom sediments of the Kłodnica River are contaminated with metals, including Pb, Zn, Ni, As, and among arsenic species, the As(V) form dominated quantitatively, with its highest concentration being 49.3 mg kg^−1^ and the organic species occurred extremely rarely. The highest concentration of arsenic, among the tested plants, occurred in *Phragmites communis* L. The evaluation of the bottom sediment pollution was performed using Sb/As factor, geoaccumulation index (*I*_geo_), enrichment factor (EF) and pollution load index (PLI). The ability of the plant to assimilate metals from the substrate was studied by calculation of the bioaccumulation factor (BAF). Values of the *I*_geo_ change in a wide range from class 1 (uncontaminated to moderately polluted for Cu and Zn) at the first sampling point, to 5 (highly to extremely polluted for Ba and Fe) at the K4 sampling point. The *I*_geo_ results show an increase in the contamination with elements toward the runoff of the Kłodnica River.

The area of Upper Silesia has been subject to strong anthropogenic pressure for many decades. The Kłodnica River flows through one of the most industrialized areas of Poland. Industrial plants, dense residential development and heavy industry are responsible for the serious pollution of surface waters. The constant drop in the groundwater level observed for many years, and more and more frequent droughts, especially in summer, often cause more than 90% of these rivers to flow with various types of sewage. Channelizing the rivers in the Kłodnica River catchment, has led to a reduction in natural floodplains (including the elimination of wetlands in river valleys), and during heavy rains coastal areas are often subject to inundation. These conditions are particularly dangerous due to the contamination of sediments by toxic elements. Along with floodwater outside the riverbed deposited sediments may enter, which is a serious threat to thousands of people living in the immediate vicinity (Jabłońska-Czapla 2015a). High concentrations of metals and metalloids in the Upper Silesia agglomeration waters have been observed for years. In the Bytomka River (a tributary of the Kłodnica River), concentrations of heavy metals (particularly Zn, Pb and Cd) often significantly exceeded the values assumed for the surface water quality classification (Jabłońska-Czapla et al. 2019). Other Kłodnica River tributaries also demonstrated increased heavy metal concentrations in water. Large-scale wastewater discharges into the Oder catchment areas already began at the beginning of the nineteenth century together with the industrial coal mining and heavy industry development (Nocoń 2006). An earlier bottom sediments study of the Odra River and its tributaries indicated a significant content of heavy metals caused by industrial and agricultural activities (Helios-Rybicka et al. 2005).

The condition of rivers, both water and bottom sediments, has a very large impact on the pollution of plants growing on the banks (Jabłońska-Czapla et al. 2020). Plants growing on the riverbanks are exposed to contaminants present in water and bottom sediment, becoming a specific indicator of ecosystem pollution. Hazardous substances penetrate coastal plants and often impair their growth. However, nature subjected to strong anthropogenic pressure in the industrialized environment has created mechanisms to protect life. Plants have developed specific mechanisms that allow them to survive in a heavily contaminated ecosystem. Inside plants, as arsenic speciation analysis showed, as can affect growth and productivity due to a plethora of morphological, physiological, biochemical, and molecular alterations (Abbas et al. 2018). Due to the ease of arsenic accumulation in plants its toxicity and plant tolerance for this element, several speciation studies were carried out in: *Acer platanoides* (Budzyńska et al. 2018), *Pteris vittata* (Wang et al. 2002) radish (*Raphanus sativus* L.) (Tlustos et al. 2002), bean (*Phaseolus vulgaris*) (Sukanya et al. 2018), *Elodea canadensis* (Picco et al. 2019), herbs (*Mentha* x *piperita*, *Matricaria recutita* (L.) *Rauschert*, *Melissa officinalis* L. and *Salvia officinalis* L.) (Jabłońska-Czapla et al. 2019). Unfortunately, there is little research in the world literature on the arsenic speciation in nettles (*Urtica dioica* L.) (Bergqvist and Greger 2012), goldenrod (*Solidago virgaurea L.*), reeds (*Phragmites communis* L.) (Tremlova et al. 2016). The literature mainly includes publications on the total metals and metalloid contents for these plants (Balabanova et al. 2015; Dyakova et al. 2018; Paukszto and Mirosławski 2019; Schulz et al. 2008).

Arsenic is a toxic metalloid common in the environment and various biological systems (Semczuk 1990). Arsenic toxicity depends not only on its total content, but also on the concentrations of its individual species. In principle, speciation is important not only in the case of arsenic, but also for many other elements (Hg, Pb, Se, etc.). Therefore, determining the total arsenic concentration is not sufficient to evaluate the risk associated with this element. As industrial pollution has not been reduced in recent decades, the arsenic emission from industry, steelworks, animal waste and dust from fossil fuel combustion is currently rising. Since arsenic is very mobile, it can be present in the environment in various chemical forms. However, its inorganic species (As(III) and As(V)) are about 100 times more toxic than its organic ones: MMA (Monomethylarsonic Acid), DMA (Dimethylarsinic Acid), AsB (Arsenobetaine) (Chen et al. 2020). For inorganic As species, arsenites are more toxic than arsenates. Therefore, determination of As speciation is essential for understanding and monitoring the fate of As in the environment. The contents of toxic species of As in the environment are still increasing, due to industrial development and economic growth (da Silva et al. 2021; Doherty et al. 2021; Johnston et al. 2020; Park and Choi 2021). In Polish rivers, the content of As(III) in water was 2.36 µg L^−1^ in the Kłodnica River (Jabłońska-Czapla 2015a) and 3.83 µg L^−1^ in the Biała Przemsza River (Jabłońska-Czapla 2015b). Contact with arsenic in humans can cause various detrimental health effects, such as dermatological, pulmonary, cardiological, genetic, genotoxic or mutagenic (Selene et al. 2003). For humans, water and food are the main arsenic sources. When compared to its inorganic forms, the organic compounds of As are relatively non-toxic to humans. Inorganic arsenic forms are metabolized in the human body to their methylated species (in the methylation process) and removed at least partly with urine (Vahidnia et al. 2007).

The application of hyphenated techniques such as high-performance-liquid chromatography-inductively coupled plasma-mass spectrometry (HPLC-ICP-MS) allows for speciation analysis (Cai et al. 2017; Donner et al. 2017; Hong et al. 2018; Marcinkowska et al. 2016). It is necessary for the hyphenated methods used in the arsenic speciation analytics (at low concentration levels) to be both appropriately selective and sensitive. There are many studies in the literature on the instrumental methods used for the speciation of arsenic chemical species. Most of them are based on chromatographic separation techniques, such as HPLC (Asaoka et al. 2012; Mir et al. 2007; Ronkart et al. 2007).

The paper presents the results of physicochemical tests of bottom sediments, water, and three plants species (*Solidago virgaurea* L., *Phragmites* L. and *Urtica dioica* L.) growing on the banks of the Kłodnica River. These materials were tested for metal(loid)s in their arsenic concentration, including the organic and inorganic arsenic species As(III), As(V), AsB, MMA and DMA using HPLC-ICP-MS techniques. The study aimed to investigate the impact of water and bottom sediment pollution of the Kłodnica River on the pollution of coastal plants such as *Solidago virgaurea* L., *Phragmites* L. and *Urtica dioica* L.

The pollution load index (PLI, Tomlinson et al. 1980), geoaccumulation index (I_geo_, Müller 1969), enrichment factor (EF, Zhang et al. 2007) and Sb/As factor (Bi et al. 2011; Fu et al. 2011; Sharifi et al. 2016) were applied to quantify the degree and origin of bottom sediment contamination by heavy metals. The ability of the plants to assimilate metals from the substrate was studied by calculation of bioaccumulation factor (BAF) (Pachura et al. 2016).

## Materials and Methods

### Research Area

The Kłodnica River is a right tributary of Odra River (Nocoń 2006). It flows through the Upper Silesia region, which is an area that is heavily urbanized and industrialized. The length of the river is 84 km, and the total catchment area is 1,125.8 km^2^. In the upper part, the river flows through the following large cities: Katowice City (K1 sampling point), Ruda Śląska City (K2 sampling point), Mikołów City, Świetochłowice City, Zabrze City and Gliwice City (K3 and K4 sampling points). In its catchment area, there are many industrial plants, including a hard coal mine. The catchment area of the river in this section is industrial. The lower section of the river from the dam to the mouth of Dzierżno Duże and in Kedzierzyn Koźle has an agricultural character (K5 and K6 sampling points). Kłodnica River from the source is contaminated with industrial wastewater and municipal waste. The content of heavy metals in sediments is much higher than in river sediments from other Polish regions with untreated strong anthropogenic pressure. The main reason for this is untreated rainfall and snowmelt from urban and industrial areas, surface runoff from landfill waste dumps and sediments lying at the bottom of Kłodnica. For years, the Kłodnica River has been a receiver of waters from the drainage of hard coal mines: Śląsk, Polska-Wirek, Bielszowice, Halemba, Makoszowy, Centrum, Bobrek, Sośnica and Gliwice. Hence, a serious problem is also the pollution of the river's ecosystem with salts from the drainage of mines. Household and municipal sewage as well as rainwater from heavily channelized Katowice, Mikołów, Ruda Śląska, Zabrze, Gliwice and Bytom has been reaching it.

### Sampling

**Fig. 1:**
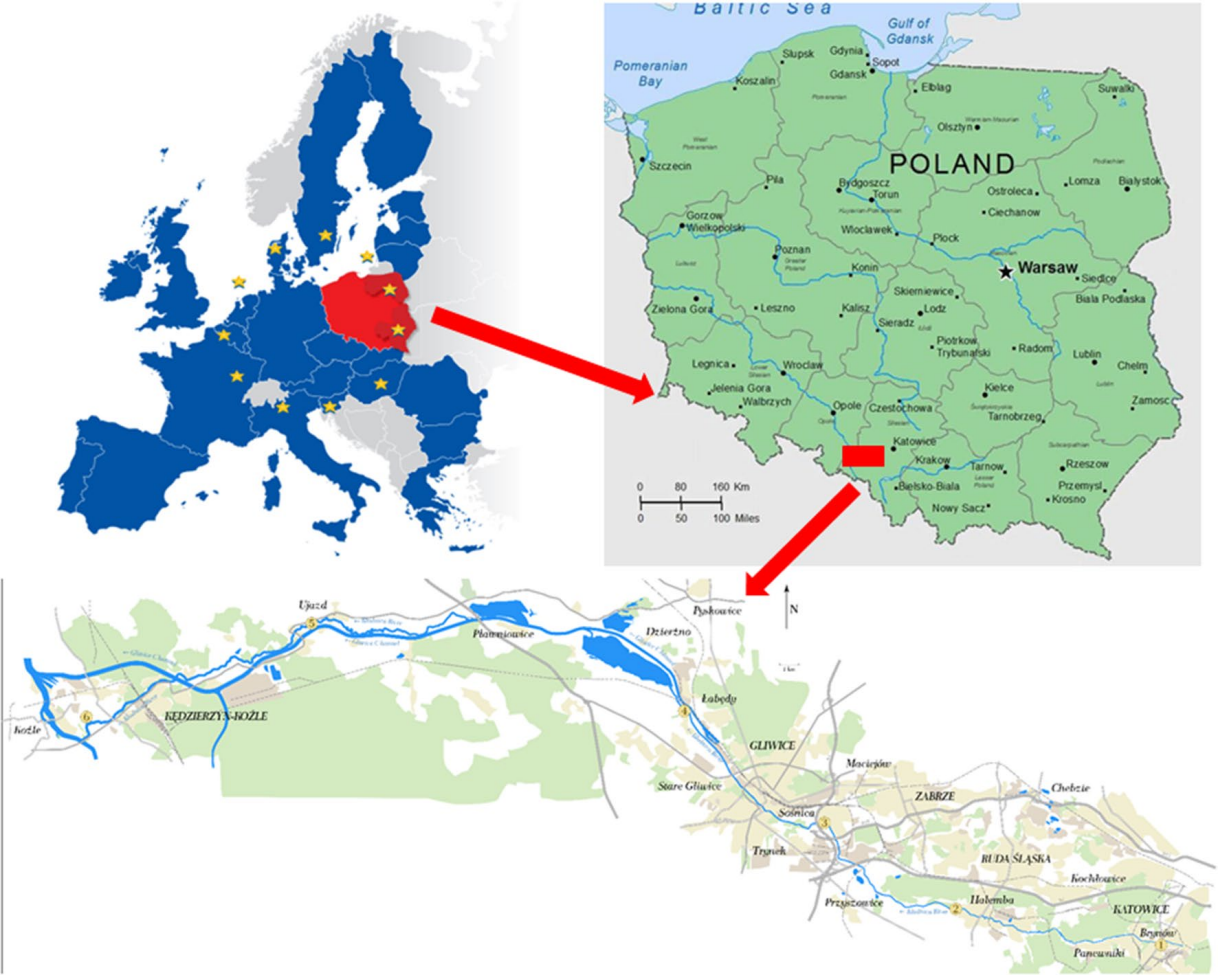
Water, bottom sediment and plant sampling points of the Kłodnica River, K1-K6 sampling points: K1 (Katowice, Warzywna Street), K2 (Paniówki the bridge), K3 (Gliwice Odrowążów Street), K4 (Gliwice Zamkowa Street) K5 (Ujazd) and K6 (Kędzierzyn Koźle)

Water and sediment samples were collected all year, monthly from April to March at the six sampling points shown in Fig. 1 and carried out similarly as in the previous study (Jabłońska-Czapla 2015b). Water samples were taken from the middle part of the river current, dipping a sampler below the water surface. Both water and bottom sediments were collected always in the same sampling points. Sediments were collected from the layer thickness of 0–5 cm. Directly after sampling in situ, basic physicochemical parameters (pH, Eh, conductivity and temperature) were determined. Plant samples of *Urtica dioica* L., *Solidago virgaurea* L., and *Phragmites* L*.* were collected in July from the six sampling points (the same as water and bottom sediment) shown in Fig. 1.

### Sample Preparation and Research Methodology

#### Water and Bottom Sediment Samples

Directly after being transported into the laboratory, the water samples were acidified with spectral grade nitric acid (V) and determined with an ICP-MS spectrometer. Afterwards, they were filtered through a 0.22-µm PES syringe filter.

After being averaged, the bottom sediment samples were air-dried, sieved through a sieve (2-mm mesh) and ground in a mortar. The sieve analysis of bottom sediments was performed following the Polish standard (Geotechnical studies 2015). Next, they were digested in the Mars X microwave oven (CEM Corporation, Matthews, NC, USA). The most effective digestion was obtained when a mixture of 5 mL of 65% HNO_3_ (Merck, Darmstadt, Germany), 3 mL of 40% HF (Merck Darmstadt, Germany) and 2 mL of H_2_O_2_ (Merck Darmstadt, Germany) were used. The total arsenic concentration was determined in water and bottom sediment digests with the ICP-MS spectrometer.

The water and bottom sediment samples for arsenic speciation analyses were stored at − 22 °C in a freezer (less than a month). The water samples were filtered through an injection PES filter (0.22 µm) just before the analysis. Afterwards, the samples were analyzed with HPLC-ICP-MS technique. The first stage of the bottom sediment sample preparation consisted of defrosting, centrifuging and extracting each sample. The extraction aimed to wash out the easily-leachable arsenic fractions from the bottom sediment samples. The extraction with the phosphate buffer in an ultrasonic bath (Jabłońska-Czapla et al. 2014a, b) for arsenic speciation was used. The simplified BCR three-step sequential chemical extraction (Tokalioglu et al. 2003) helped to determine the arsenic forms in the bottom sediments and how they were bound. The conditions under which the sequential extraction was performed are given in (Jabłońska-Czapla 2015b).

#### Plant Samples

After being transported to the laboratory, the collected plant samples were washed with deionized water, cut, dried in the air and ground in a porcelain mortar to determine the total elements including arsenic concentration. Then the plant samples were combined with 5 mL spectral HNO_3_ and 3 mL water, and digested in a MarsX microwave oven (digestion power – 600 W, for 5 min up to 100 °C, than 600 W for 15 min up to 150 °C and a stop time of 10 min), and then the arsenic content was determined using an ICP-MS spectrometer.

The plant samples for speciation analyses were washed with deionized water, cut and stored at − 22 °C in a freezer for no longer than a month. Based on in-depth literature analysis, the extraction of plant samples was not optimized. However, a method offering the highest arsenic extraction yield from the samples of the plant origin was selected (Zheng et al. 2003). A 1-g sample of plant material was extracted with 10 mL of water:methanol solution in a 1:9 ratio. HPLC-purity methanol was used. Each plant sample was tested in triplicate and extracted with 2-h shaking in a shaker (165 rpm). The extracted plant samples underwent filtration through a 0.22-μm syringe filter and were injected into the HPLC-ICP-MS system for the arsenic speciation analysis.

#### Reagents and Standard Solutions

The in situ physicochemical data temperature, pH (PN-EN ISO 10523:^2012^) electrolytic conductivity (EC) (PN EN 27888:^1999^) and redox potential (Eh) were measured using the CX-401 multi-parameter meter (Elmetron, Poland) equipped with an ERH 111 glass electrode (Hydromet, Poland), an ERPt-111 platinum electrode (Hydromet, Poland) and a CD-2 conductometric sensor (Hydromet, Poland) with a built-in thermometer. The EC and pH values were double-checked, and the pH meter was calibrated with three pH buffer solutions (pH = 3.0, 7.0, and 9.0).

The Elan 6100 DRC-e ICP-MS spectrometer (Perkin Elmer) was used for quantitative analyses of total arsenic content in water, digested sediment and plant samples. The apparatus was equipped with a standard ICP quartz torch, cross-flow nebulizer and nickel cones. Samples and standards were delivered with a peristaltic pump. The spectrometer was optimized daily with a 10-µg L^−1^ solution (Mg, Cu, Rh, Cd, In, Ba, Ce, Pb and U) in 1% HNO_3_ Elan 6100 Setup/Stab./Masscal. Solution (Perkin Elmer). The concentration of ^75^As and other elements (^51^ V, ^53^Cr,^55^Mn, ^57^Fe, ^59^Co, ^60^Ni, ^65^Cu, ^66^Zn, ^69^ Ga,^85^Rb, ^88^Sr, ^98^Mo,^107^Ag, ^114^Cd, ^121^Sb, ^126^Te, ^138^Ba, ^205^Tl, ^208^Pb, ^238^U) was measured with the internal ^103^Rh standard. The multi-elemental standards no. XXI and VI (Merck, Germany) were used when determining total arsenic concentration with ICP-MS.

The arsenic (As(III), As(V), AsB, MMA, and DMA) species were determined with the HPLC-ICP-MS system. To separate the analytes, a speciation apparatus set was applied. It consisted of the HPLC chromatograph (Perkin Elmer) equipped with a Series 200 LC Peltier oven, Series 200 LC autosampler and Series 200 LC gradient pump. The operating parameters of the ICP-MS spectrometer are given in the study (Jabłońska-Czapla 2015b). The HPLC-ICP-MS separation was performed after optimization and applied a Hamilton PRP-X100 (150 mm × 4.6 mm, 5 µm) column at a temperature of 30 °C, with solutions A – (20 mM NH_4_NO_3_, pH = 8.7) and B – (60 mM NH_4_NO_3_, pH = 8.7) as the mobile phase, and the elution programme was (0–2.0 min 100% A, 2.0–3.0 min from 100% A to100% B, 3.0–6.5 min 100% B, rinsing 6.5–9.5 min 100% A). The flow rate during the analysis and rinsing was 1.1 mL min^−1^ and the volume of the sample was 100 µL. The retention times were as follows: AB 1.84 min, As(III) 2.16 min, DMA 2.95 min, MMA 5.36 min and As(V) 6.17 min (Jabłońska-Czapla and Szopa 2016).

The following substances were used for analyses: ultra-pure ammonium nitrate (Merck, Darmstadt, Germany); sodium dihydrogen arsenate heptahydrate (Sigma-Aldrich, St Louis, MO, USA); sodium arsenite (Sigma-Aldrich, St Louis, MO, USA); disodium methyl arsenate (Supelco, Bellefonte, PA, USA); arsenobetaine (Sigma-Aldrich, St Louis, MO, USA); dimethylarsinic acid (Supelco, USA), ultrapure nitric acid (65%, Merck, Germany), analytically pure dihydrogen potassium phosphate (POCH, Poland), analytically pure disodium phosphate (POCH, Poland) and HPLC isocratic grade methanol (J.T. Baker, Netherlands).

The calibration solutions were prepared each time by diluting suitable standard solutions on an analytical balance. The multi-element standards no. XXI and VI (Merck, Darmstadt, Germany) were used for determining total metal(loid) contents with the ICP-MS spectrometer. Solutions made from salts were used for calibration during quantitative determinations of the As species. All solutions and standards were prepared with Milli-Q-Gradient ultra-pure deionized water (Millipore, Merck Darmstadt, Germany), whose electrolytic conductivity was < 0.05 μS cm^−1^.

#### Quality Control

The method validation was performed based on the certified reference materials (NIST 1643e Trace Elements in Water, NCS DC 73309 for bottom sediments, NIST Tomato Leaves CRM 1573a for plants). The arsenic concentrations were determined with a 0.096 µg L^−1^ detection limit. The recovery and the extraction efficiency of total arsenic concentration in plants (water/methanol extraction efficiency 9:1) were checked using the certified reference material NIST Tomato Leaves 1573a, and it was 41.7%. To check the recovery of the proposed extraction procedure of arsenic species, the standard addition method was used.

During the determination of arsenic species (As(III), As(V), AB, MMA, DMA), the following LODs (limits of detection) were obtained: 0.08 µg L^−1^, 0.12 µg L^−1^, 0.16 µg L^−1^, 0.08 µg L^−1^ and 0.09 µg L^−1^, respectively. The analyses were characterized by a low relative standard deviation of repeatability and amounted to As(III) 2.9%, As(V) 2.4%, AB 3.7%, MMA 3.1% and DMA 2.7%. The recovery percentages of arsenic species (As(III), As(V), AB, MMA, DMA) were 96%, 104%, 93%, 95% and 95%, respectively.

#### Assessment of the Kłodnica River Sediment and Coastal Plant Contamination

The evaluation of the bottom sediments pollution was performed using Sb/As a factor, which is one of the indicators of pollution origin sources (Bi et al. 2011; Fu et al. 2011; Sharifi et al. 2016), along with the geoaccumulation index (*I*_geo_) proposed by Müller (1969), the enrichment factor (EF) (Zhang et al. 2007) and the pollution load index (Tomlinson et al. 1980).

The geoaccumulation index (*I*_geo_) is defined as:

1 $$ I_{{{\text{geo}}}} = \log_{2} \left( {C_{{{\text{EL}}}} /1.5 \, C_{{{\text{background}}}} } \right) $$

where *C*_EL_ is the total element concentration in the bottom sediment samples, *C*_background_ is the geochemical background of element concentration, and factor 1.5 is a correction factor compensating for natural (lithological) variations in geochemical data. *I*_geo_ describes the pollution of the bottom sediments by an element concerning seven classes from 0 to 6.

If *I*_geo_ ≤ 0, the class is unpolluted; if 0 < *I*_geo_ ≤ 1, the class is from unpolluted to moderately polluted; if 1 < *I*_geo_ ≤ 2, the class is moderately polluted; if 2 < *I*_geo_ < 3, the class is from moderately polluted to strongly polluted; if 3 < *I*_geo_ < 4, the class is strongly polluted; if 4 < *I*_geo_ < 5, the class is from strongly polluted to extremely polluted and *I*_geo_ > 5 means extremely polluted.

The pollution load index (PLI) is often used to determine the level of the bottom sediments contamination compared to background concentrations levels (Lis and Pasieczna 1998). To calculate the PLI, the following equations were used:

2 $$ C_{{{\text{FEL}}}} = \, C_{{{\text{EL}}}} /C_{{{\text{background}}}} $$

3 $$ {\text{PLI}} = \left( {C_{{{\text{FEL}}1}} \times C_{{{\text{FEL}}2}} \times C_{{{\text{FEL}}3}} \times \cdots \times C_{{{\text{FEL}}n}} } \right)^{1/n} $$

where *C*_FEL_ is the pollution factor defined as the ratio of the element concentration in the bottom sediment to the concentration of this element in the background.

In our case, we used the metal enrichment factor (EF) as an index to evaluate anthropogenic influences of heavy metals in sediments, and we can reasonably evaluate the metal contamination in the area. According to Zhang et al. (2007), the metal enrichment factor (EF) is defined as follows:

4 $$ {\text{EF}} = \left( {{\text{Me}}/{\text{Fe}}} \right)_{{{\text{sample}}}} /\left( {{\text{Me}}/{\text{Fe}}} \right)_{{{\text{background}}}} $$

where the (Me/Fe)_sample_ is the metal-to-Fe ratio in the samples of interest and the (Me/Fe)_background_ is the natural background value of the metal to Fe ratio. If the EF is 0.5 ≤ EF ≤ 1.5, the trace metal originates from a crustal source. If EF > 1.5, it indicates the increasing contribution of non-crustal (biota and/or pollution) sources.

One of the important parameters to determine the ability of the plant to assimilate metals from a substrate is the bioaccumulation factor (BAF). It is defined as the ratio of the content of the given metal of the plant to its total content in the sediment. According to following formula, the BAF was calculated separately for all three plant species in each sampling point.

5 $$ {\text{BAF}} = \left( {{\text{MC}}_{{{\text{plant}}}} } \right)/\left( {{\text{MC}}_{{{\text{substrate}}}} } \right) $$

where BAF is the bioaccumulation factor, MC_plant_ is the metal concentration in plant (all parts) in mg kg^−1^ and MC_substrate_ is the metal concentration in sediment in mg kg^−1^.

A four-degree scale was used to assess the transfer of metals to the plants: no accumulation (< 0.01), low (0.01–0.1), medium (0.1–1.0) and high (> 1.0) (Pachura et al. 2016).

## Results and Discussion

### Physicochemical Parameters

**Fig. 2:**
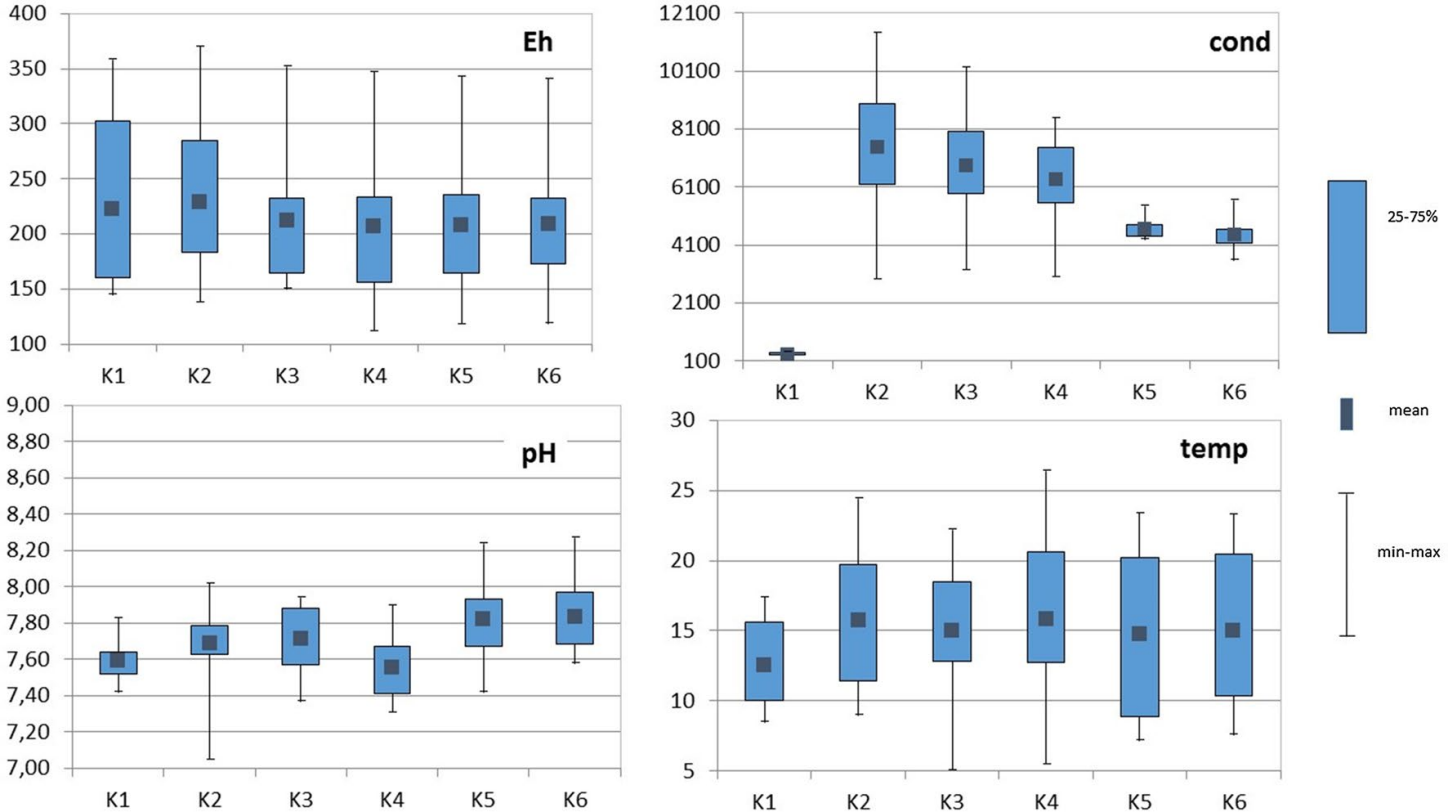
Box plots of pH, conductivity (cond in μS), temperature (temp in °C) and redox potential (Eh) data for the Kłodnica River monitoring; sampling points K1 (Katowice, Warzywna Street), K2 (Paniówki the bridge), K3 (Gliwice Odrowążów Street), K4 (Gliwice Zamkowa Street), K5 (Ujazd) and K6 (Kędzierzyn Koźle)

The results show an increase in the pH of water along the river course (Fig. 2). Exceptionally, the pH was decreased at the K4 sampling point. The reason for this may be its proximity to the sewage treatment plant, which is likely to affect the pH of the water in the Kłodnica River. Similar results were obtained previously in 2004 (Nocoń 2006). The strong salinity of the Kłodnica River affects the high water conductivity in the central part of the agglomeration at the K2-K4 sampling points. At the first sampling point (K1), at the source of the Kłodnica River flowing out of the Murckowski Forests, the water is characterized by low conductivity, which is a sign of low salinity. At the K5 and K6 points, the physicochemical parameters of the Kłodnica River water improved, there are no major industrial plants, and the Kłodnica River itself is subjected to the agricultural anthropogenic pressure head. The highest conductivity and redox potential was observed in May and June. Interestingly, in July, a significant drop in these physicochemical parameters was observed.

This may be related to heavy rainfall occurring at this time. The extraction from deep reservoirs in the preindustrial world was primarily due to volcanoes and oceanic hydrothermal inputs. However, more recently, mining activity (e.g., metal ore extraction and processing) as well as the extraction of coal and hydrocarbon production have greatly exacerbated the inputs of metal(loid)s to aquatic ecosystems. Additionally, metal(loid)s are also reduced during the consumption of these products, especially through coal and hydrocarbon burning for energy and transportation. The sources of metal(loid)s to freshwaters (rivers and lakes) are the erosion of surface terrestrial material, the impute of metal(loid)s associated with groundwater and runoff and the deposition of metal(loid)s from the atmosphere (Mason 2013). Taking into account the level of Silesia air pollution, the effect is quite large (Rogula-Kozłowska et al. 2013, 2015).

### Total Metal(loid)s Content in Water, Sediments and Plants

The sieve analysis of the Kłodnica River bottom sediments (Table 1) indicated that, at the first and second sampling points, the bottom sediment consisted mainly of coarse and medium sand, and then at sampling points K3 and K4, the bottom sediment was finer and comprised mainly of medium and fine sand. Along the course of the Kłodnica River, its condition improved in quality, which was also visible in an increase in the proportion of coarse sand in the bottom sediment at the fifth sampling point (K5). At the last collection point, just before the mouth of the Kłodnica River to the Odra River, the bottom sediment consisted mainly of gravel and coarse to medium sand.

**Table 1:** The sieve analysis of the Kłodnica River bottom sediments, sampling points: K1 (Katowice), K2 (Ruda Śląska), K3 (Gliwice Odrowążów Street), K4 (Gliwice Zamkowa Street), K5 (Ujazd) and K6 (Kędzierzyn Koźle)

Sampling Point	> 2.0 mm	2.0–1.0 mm	1.0–0.5 mm	0.5–0.2 mm	0.2–0.1 mm	< 0.1 mm	Unit
K1	3.86	4.32	35.7	52.4	2.62	0.92	%
K2	1.15	2.08	19.5	68.4	8.68	0.09	%
K3	4.68	9.58	6.69	23.2	31.2	24.1	%
K4	22.6	9.37	6.94	18.0	19.9	22.9	%
K5	1.92	5.05	25.4	66.3	1.18	0.02	%
K6	22.0	24.0	40.1	13.8	0.03	0.02	%

Tables 2 and 3 show the total metal and metalloid contents in the water, plant and bottom sediments of the Kłodnica River. In terms of the metal and metalloid contents, the Kłodnica River meets the Polish regulations about the classification of surface waters and environmental quality standards for priority substances (Regulation of Minister of Environment 2016), as well as guidelines for drinking water (Regulation of the Minister of Health 2017). The Kłodnica River water flowing through the central part of Upper Silesia contained elevated metal contents, with the highest concentrations of Ni, Zn, Rb, Sr or Pb occurring in water sampled from the K2-K4 points. The Kłodnica River flowing through the less industrialized part of Silesia is self-cleaning, which is visible in the form of lower concentrations of metals and metalloids. The concentration of arsenic in the waters did not exceed 11 µg L^−1^. This elemental contamination of bottom sediments following the geochemical classification (Siebielec et al. 2015) was significant, and the bottom sediments of the Kłodnica River should be classified as class II for surface water purity. The highest concentration of arsenic in the bottom sediment occurred in K4 sampling point and was equal to 17.0 mg kg^−1^. Moreover, at this point, the bottom sediments of the Kłodnica River are heavily polluted by Cd, Cr, Cu and Pb. Due to the concentration of these elements, following the geochemical classification, bottom sediments may be classified as the II class of purity. The greatest bottom sediment contamination occurred at the third (K3) and fourth (K4) sampling points. At the fourth sampling point, the lead concentration was, on average 188 mg kg^−1^. Even higher results for metal contents in the bottom sediment were obtained for zinc, barium and silver. For these elements, the contamination of the bottom sediments of the Kłodnica River was so high that the bottom sediments should be included in the third class of purity. In Gliwice (K4), the concentrations of zinc and barium were 1302 mg.kg^−1^ and 3304 mg kg^−1^, respectively.

**Table 2:** Mean total elements concentration in water (W, number of replicates *n* = 12) and bottom sediment (BS, number of replicates *n* = 12) of the Kłodnica River; K1-K6 sampling points; validation parameters: LOD (limit of detection µg L^−1^), U (uncertainty, %), R (recovery of CRM bottom sediment NCS DC 73309, %)

Element	Validation parameters			K1		K2		K3		K4		K5		K6	
	LOD	U [%]	R[%]	BS	W	BS	W	BS	W	BS	W	BS	W	BS	W
V	0.09	10.00	111	7.03	1.04	8.19	12.6	23.0	12.8	86.6	12.2	3.85	8.69	8.74	7.93
Mn	0.033	21.08	98	365	121	92.8	347	333	269	574	378	176	301	644	304
Co	0.002	10.00	96	2.12	0.31	1.99	2.00	4.29	1.44	10.9	1.61	1.40	1.08	3.23	1.04
Ni	0.024	10.00	103	4.82	2.48	3.74	8.81	13.4	10.8	29.7	11.5	4.48	8.94	5.77	8.65
Cu	0.064	12.37	100	10.5	1.74	7.21	4.82	27.2	4.36	73.6	4,41	6.01	3.95	11.2	3.82
Zn	0.181	34.65	83	214	84.6	338	51.43	611	58.0	1302	47.9	307	21.9	124	25.8
As	0.096	27.81	101	1.61	0.74	2.34	10.15	7.09	10.7	17.9	9.52	2.60	6.89	3.17	6.29
Rb	0.003	10.00	132	18.2	3.37	17.5	45.7	25.6	43.8	29.7	40.9	26.5	27.6	20.5	25.5
Sr	0.008	10.62	81	20.2	158	22.7	2351	68.3	2781	158	2469	24.8	1798	32.2	1694
Ag	0.002	14.05	108	0.34	0.08	6.76	0.27	1.45	0.09	1.86	0.07	3.56	0.04	0.57	0.04
Cd	0.04	11.38	80	3.54	0.62	3.02	0.29	2.71	0.32	2.28	0.23	0.29	0.09	0.33	0.12
Te	0.002	12.65	108	1.18	0.70	1.27	0.93	3.22	0.73	22.1	0.83	1.49	0.81	1.59	0.83
Ba	0.01	16.96	67	161	61.6	188	79.6	427	63.9	3304	70.7	202	67.7	202	72.4
Tl	0.002	15.18	118	0.21	0.20	0.76	0.08	0.79	0.14	1.17	0.08	0.21	0.08	0.83	0.18
Pb	0.036	27.98	104	21.35	1.26	16.7	2.93	152	7.30	188	4.78	18.1	1.22	21.9	1.58
Fe	0.09	31.9	115	6178	813	4558	1921	16,353	1651	56,990	1973	5429	1139	9293	1257
Ga	0.01	15.50	87	6.57	2.81	6.39	2.65	17.6	2.10	105	2.31	8.06	2.27	9.21	2.44
Mo	0.09	16.6	92	0.53	0.52	0.49	2.26	0.70	3.75	1.89	3.44	0.19	3.62	1.74	3.33
U	0.01	11.39	95	0.47	1.49	0.73	1.16	1.07	0.10	2.89	0.38	0.44	0.79	0.81	0.83
Cr	0.013	11.44	89	8.19	2.63	12.08	41.8	31.0	41.2	57.5	39.6	4.80	29.2	17.50	27.0
Sb	0.05	10.92	101	0.48	0.30	0.45	1.30	1.47	1.09	2.90	1.13	0.46	0.91	0.53	0.83

*CRM* Certificated Reference Material

**Table 3:** Mean total elements concentration in plants (number of replicates *n* = 3): *Urtica dioica* L. (Ud, µg g^−1^), *Solidago virgaurea* L. (S, µg g^−1^), *Phragmites communis* L*.* (P, µg g^−1^) from the Kłodnica River bank; K1-K6 sampling points

E	K1			K2			K3			K4			K5			K6		
	*Ud*	*S*	*P*	*Ud*	*S*	*P*	*Ud*	*S*	*P*	*Ud*	*S*	*P*	*Ud*	*S*	*P*	*Ud*	*S*	*P*
V	0.76	0.46	1.68	1.13	0.37	1.73	0.47	0.26	1.01	0.16	1.18	0.68	0.55	0.19	0.54	0.39	0.11	0.40
Mn	53.5	25.5	1.42	56.1	25.6	0.20	0.11	40.1	43.7	30.5	35.8	37.5	53.8	0.11	21.2	26.9	37.1	53.5
Co	0.21	0.21	1.77	0.60	0.15	1.13	0.16	0.16	0.29	0.07	0.33	0.22	0.20	0.14	0.11	0.20	0.07	0.13
Ni	3.30	0.73	8.24	5.10	0.86	18.38	2.80	1.34	1.65	1.65	3.90	2.51	0.46	3.47	2.43	1.46	1.77	1.99
Cu	7.34	9.37	11.0	8.25	7.48	8.23	9.66	10.9	8.48	14.5	12.05	8.38	6.23	5.47	5.98	8.95	6.55	4.42
Zn	50.3	56.06	173	36.74	57.5	49.9	78.1	51.2	65.7	75.1	65.4	44.9	19.8	25.5	32.9	46.3	15.0	21.0
As	0.30	0.18	0.70	0.37	0.15	0.50	0.18	0.10	0.45	0.09	0.33	0.27	0.19	0.07	0.19	0.12	0.05	0.14
Rb	16.8	25.3	34.8	19.7	26.5	12.2	21.4	24.7	10.7	32.0	17.87	14.5	10.6	5.28	14.9	5.35	9.13	12.08
Sr	65.3	19.8	16.9	188	0.20	0.15	3.18	30.6	173	26.7	18.8	108	136	0.31	111	27.8	25.5	112
Ag	0.07	0.01	1.23	0.18	0.03	0.09	0.09	0.02	0.14	0.04	0.05	0.14	0.04	0.06	0.14	0.01	0.01	0.10
Cd	0.76	1.62	1.75	0.22	0.95	0.59	0.09	0.67	0.20	0.38	0.22	0.13	0.08	0.05	0.12	0.57	< 0.04	0.06
Te	0.47	0.16	0.45	0.60	0.03	0.64	0.19	0.07	0.19	0.01	0.21	0.30	0.20	0.53	0.27	0.10	0.09	0.18
Ba	46.5	15.3	6.36	105	5.15	0.52	20.7	5.71	17.7	2.42	21.9	30.4	20.0	0.76	18.9	12.1	12.2	17.2
Tl	0.12	0.10	0.04	0.62	0.24	0.02	0.11	0.48	0.73	0.20	0.05	0.27	0.06	0.11	0.18	0.09	0.15	0.09
Pb	5.03	1.43	5.86	4.23	0.78	8.92	2.60	1.02	4.26	0.16	4.21	3.04	1.51	26.8	1.38	1.01	0.48	0.83
Fe	466	239	763	1827	187	15.04	316	152	570	162	655	525	522	520	472	325	162	295
Ga	1.60	0.52	2.01	2.18	0.25	2.88	0.93	0.19	0.63	0.10	1.03	0.99	0.67	2.26	0.78	0.53	0.47	0.57
Mo	1.37	0.48	0.89	1.27	0.05	1.02	0.34	0.14	0.67	0.23	1.14	0.67	0.83	1.12	0.68	0.05	0.41	0.78
U	0.02	< 0.01	0.09	0.04	0.01	0.05	0.01	0.02	0.02	< 0.01	0.04	0.03	0.01	0.01	0.02	0.02	0.01	0.02
Cr	6.89	4.06	18.5	20.9	0.95	63.9	6.10	2.48	3.83	0.82	16.0	7.25	5.81	8.38	6.39	1.95	3.78	5.03
Sb	0.22	0.08	0.22	0.08	< 0.05	0.14	< 0.05	< 0.05	0.11	< 0.05	0.07	< 0.05	< 0.05	< 0.05	0.05	< 0.05	< 0.05	0.06

Low metal and metalloid concentrations in the Kłodnica River water clearly indicated that the condition of this river has improved in recent years, while the high concentrations of deposited substances in bottom sediments are an indicator of long-term anthropogenic pressure pollution. In addition, as shown in Table 2, concentrations of elements such as lead are reduced about 10 times in bottom sediments collected at points outside the central part of the Upper Silesian agglomeration in Ujazd and Kędzierzyn Koźle (K5 and K6).

### Sequential Chemical Extraction of Bottom Sediments

The modified BCR (the Institute for Reference Materials and Measurements, Tokalioglu et al. 2003) extraction results show that arsenic was mainly bound to the immobile oxidizable fraction (associated with organic substance and sulfides) and at a lower level with a mobile reducible fraction (associated with iron/manganese oxides) in the Kłodnica River bottom sediments (Fig. 2). Arsenic was quite strongly demobilized in the Bytomka River bottom sediments. At the K3 sampling point, the proportion of arsenic in the ion exchange fraction was the highest at 12%, similar to the mobile fraction associated with oxides (35%). According to other authors, As was mainly bound to iron hydroxides and organic/sulfide fraction in river bottom sediments (Ciszewski 2003; Jabłońska-Czapla 2015b). The bottom sediment research in the Kłodnica River confirmed this information. The arsenic was the most demobilized at the fourth bottom sediment collection point. Metals bound to the ion exchange and carbonate fractions (mobile fraction) are the main environmental hazards as they can easily migrate to the solution, which makes them more bioavailable and potentially toxic (Tokalioglu et al. 2003). Conversely, metals bound to the organic and aluminosilicate fractions are considered less bioavailable due to the low probability of their release into the solution. From a biological viewpoint, metals/metalloids bound to the ion-exchange fraction are the most harmful due to their bioavailability. Due to the surface water self-purification processes, dissolved heavy metal forms are transported into the bottom sediments during sorption and other biochemical processes. Consequently, the water quality could be improved, but the metal concentrations in the bottom sediments would be increased. The heavy metal content in the bottom sediments is a good indicator of the water environment pollution level (Giri and Singh 2014).

### Arsenic Species in Plants

Table 4 presents the content of arsenic species in the tested plants growing on the Kłodnica River banks. The *Phragmites communis* L. was characterized by the highest arsenic content. At the K1-K3 sampling points (the centre of the Upper Silesian Industrial District), the *Phragmites communis L.*, apart from As(III) and As(V), also contained methyl arsenic derivatives. In turn, *Urtica dioica* L. and *Solidago virgaurea* L*.* were characterized by the presence of organic arsenic forms also outside the industrial region, in areas subjected to agricultural anthropogenic pressure. All tested plants contained inorganic arsenic forms, mainly As(V), as well as MMA and/or DMA. Research has shown the absence of AsB in the above-mentioned marginal plants of the Kłodnica River. The research results of the arsenic species content in coastal plants (*Urtica dioica* L., *Galium aparine* L., *Potamogeton pectinatus*) of the Bytomka River (which is a tributary of the Kłodnica River) have also shown that plants contain the largest amounts of As(V) and As(III) as well as methyl derivatives such as MMA and DMA. AsB was marked only in one plant sample (Jabłońska-Czapla et al. 2020).

**Table 4:** Arsenic species: As(V), As(III), MMA, DMA, AB in plants, water and bottom sediment of the Kłodnica River; K1-K6 sampling points

Sampling points	*Urtica dioica* L	*Solidago virgaurea* L	*Phragmites communis* L	Water	Bottom sediment
	[µg g^−1^]			[µg L^−1^]	[µg g^−1^]
K1	As(V) 0.18	As(V) 0.13	As(V) 0.36	As(V) 0.46	As(V) 1.03
	As(III) < 0.08	As(III) < 0.08	As(III) < 0.08	As(III) 0.17	As(III) 3.35
	DMA < 0.09	DMA < 0.09	DMA < 0.09	DMA < 0.09	DMA < 0.09
	MMA 0.012	MMA < 0.08	MMA 0.08	MMA 0.11	MMA 0.09
	AB < 0.16	AB < 0.16	AB < 0.16	AB < 0.16	AB < 0.16
K2	As(V) 0.64	As(V) 0.27	As(V)0.12	As(V) 8.85	As(V) 3.17
	As(III) < 0.08	As(III) 0.004	As(III) 0.09	As(III) < 0.08	As(III) 0.18
	DMA < 0.09	DMA < 0.09	DMA0.09	DMA < 0.09	DMA < 0.09
	MMA < 0.08	MMA 0.12	MMA < 0.08	MMA < 0.08	MMA < 0.08
	AB < 0.16	AB 0.20	AB < 0.16	AB < 0.16	AB < 0.16
K3	As(V) 0.28	As(V) 0.57	As(V) 0.26	As(V) 5.44	As(V) 1.00
	As(III) 0.01	As(III) 0.01	As(III) 0.018	As(III) 1.22	As(III) 0.93
	DMA < 0.09	DMA < 0.09	DMA < 0.09	DMA < 0.09	DMA < 0.09
	MMA 0.10	MMA 0.08	MMA 0.09	MMA < 0.08	MMA 0.09
	AB < 0.16	AB < 0.16	AB < 0.16	AB < 0.16	AB < 0.16
K4	As(V) 0.12	As(V) 0.35	As(V) 0.13	As(V) 4.08	As(V) 49.3
	As(III) 0.08	As(III) 0.09	As(III) 0.12	As(III) 0.18	As(III) 6.82
	DMA 0.09	DMA 0.18	DMA < 0.09	DMA < 0.09	DMA < 0.09
	MMA 0.16	MMA < 0.08	MMA < 0.08	MMA < 0.08	MMA < 0.08
	AB < 0.16	AB < 0.16	AB < 0.16	AB < 0.16	AB < 0.16
K5	As(V) 0.12	As(V) 0.26	As(V) 0.14	As(V) 5.11	As(V) 13.2
	As(III) 0.25	As(III) < 0.08	As(III) 0.22	As(III) 1.10	As(III) 9.35
	DMA < 0.09	DMA < 0.09	DMA < 0.09	DMA < 0.09	DMA < 0.09
	MMA 0.09	MMA 0.32	MMA < 0.08	MMA < 0.08	MMA < 0.08
	AB < 0.16	AB < 0.16	AB < 0.16	AB < 0.16	AB < 0.16
K6	As(V) 0.13	As(V) 0.45	As(V) 0.90	As(V) 3.52	As(V) 1.75
	As(III) < 0.08	As(III)0.046	As(III) < 0.08	As(III) 1.56	As(III) 1.96
	DMA < 0.09	DMA < 0.09	DMA < 0.09	DMA < 0.09	DMA < 0.09
	MMA < 0.08	MMA < 0.08	MMA < 0.08	MMA < 0.08	MMA 0.08
	AB < 0.16	AB < 0.16	AB < 0.16	AB < 0.16	AB < 0.16

Plants partially metabolize arsenic into its methyl derivatives, especially in strong pressure conditions (Jabłońska-Czapla et al. 2019). It is a kind of a defense mechanism against environmental pollution. In the methylation processes, plants transform inorganic ionic arsenic forms into MMA or DMA. The polluted environment in which the plants grow has an important impact on the content of metal(loid)s in coastal Kłodnica River plants. High arsenic content in the bottom sediment (K4, 17.9 mg kg^−1^) as well as in water (maximum 10.7 μg L^−1^) of the river over which the plants grow significantly increases the concentration of this metalloid in plants. Plants are often able to change their tolerance to a given element in the presence of elevated concentrations in the environment. The plant populations growing on polluted soils can adapt their tolerance to the current metal content in the soil (Szakova et al. 2009, 2011).

The nettles (*Urtica dioica* L.) collected on the Kłodnica River banks had a lower arsenic concentration than on the Bytomka River banks. The highest As concentration in *Urtica dioica L.* collected from the K2 sampling point was 0.37 mg kg^−1^, while the highest concentration of this element in a nettle from the Bytomka River was up to 29 mg kg^−1^.

In the Kłodnica River water and sediments, the oxidized As form was predominant; neither AsB nor DMA was found. As(V) form dominated quantitatively in the Kłodnica River sediments and its highest concentration was 49.3 mg kg^−1^. The organic species occurred extremely rarely. DMA was not found at all, while MMA was only observed in three bottom sediment samples. At the first and the sixth sampling points the As(III) concentration in the bottom sediment was slightly higher than As(V). The highest As concentration in bottom sediment (17.9 mg kg^−1^) was found at the fourth sampling point. This will affect the high arsenic content in *Solidago virgaurea* L. (0.33 µg g^−1^). However, among the tested plants, the highest concentration of arsenic occurred in *Phragmites communis* L. It is generally known that under oxidative conditions (i.e., when Eh is positive and pH < 6.9) As(V) is the dominant form. At higher pH values, As(III) (reduced form) dominates. As(V) dominates in a highly acidic environment. When pH is less than 9.2 and under reducing conditions (negative Eh), As occurs as As(III) (Selene 2003). As the redox conditions of river systems are generally oxic, the main As species in the river water and particulate matters (and sediments) are pentavalent (Jabłońska-Czapla and Zerzucha 2019).

### Metal(loid)s contamination of Kłodnica River coastal plants

The Kłodnica River water and bottom sediment contained increased amounts of arsenic. In the centre of the Upper Silesian agglomeration (K2-K4 sampling points) the concentration of arsenic in water was on average 10 µg L^−1^, and in bottom sediments it was as high as 17.9 mg kg^−1^ (K4 sampling point). The polluted ecosystem of the Kłodnica River caused an arsenic concentration in *Phragmites communis* L. of 0.70 mg kg^−1^, and in *Solidago virgaurea* L*.*, it was maximally 0.33 mg kg^−1^. Different sources of freshwater, such as lakes, rivers and streams, have As concentrations ranging from 0.15 to 0.45 µg L^−1^, depending on the sources and geochemical properties of the region (Abbas et al. 2018). When bottom sediments contain large amounts of this element, it can be taken up by plants. In all plant species tested so far, it has been shown that arsenate is taken up via the phosphate transport systems (Wang et al. 2002). Arsenate resistance has been identified in a range of plant species, which is generally achieved through a decreased uptake of arsenate because of suppression of the high-affinity phosphate uptake system.

The Kłodnica River flows through areas subjected to industrial and agricultural anthropogenic pressure. The results of the trace elements (TE) content clearly indicate the strong impact of anthropogenic pressure types on the content of elements in the waters and bottom sediments of the Kłodnica River, which in turn affects the content of these TE in coastal plants. The highest concentration of nickel in the waters and bottom sediments of the discussed river occurred at the third and fourth sampling points. This element enters the bottom sediments mainly from anthropogenic sources. Because of the increased Ni content in bottom sediments and water, 3.90 mg kg^−1^ Ni was determined in *Solidago virgaurea* L*.*, but it is not clear why in *Phragmites communis* L*.*, at the first and second sampling points, were found with 8.24 and 18.38 mg kg^−1^ Ni, respectively.

The Kłodnica River flows through a region famous for hard coal processing and mining and the metallurgy industry, so the increased content of zinc and lead in the waters and bottom sediments of this river is not surprising. The zinc concentration in bottom sediments increased along the river in such a way that the maximum concentration (1302 mg kg^−1^) of zinc in bottom sediments occurred at the fourth sampling point in Gliwice City. However, the contents of this element in plants were high at the K1-K4 sampling points for *Phragmites communis* L. (21–173 µg g^−1^), *Urtica dioica* L. (19.8–78.1 µg g^−1^) and *Solidago virgaurea* L. (15–65.4 µg g^−1^).

Similarly, in the case of lead, the content of this element was the highest at the 3rd and 4th sampling points, and the plants showed variability in the content of this element. In the case of *Phragmites communis* L., the highest Pb concentration was found at the second sampling point (8.92 mg kg^−1^), and in *Solidago virgaurea* L. the lead concentration was as much as 26.8 mg kg^−1^. The differences in the method of uptake and accumulation of heavy metals in plants are affected by the physicochemical conditions prevailing at the sampling points, and as shown in Fig. 2, they were varied.

Contamination of the Kłodnica River bottom sediments by thallium increases the content of this element in coastal plants. In the third point of K3 uptake, the mean thallium concentration in the bottom sediment was very high and amounted to 0.79 mg kg^−1^, while the concentration of this element in plants such as *Solidago virgaurea* L. and *Phragmites communis* L*.* were 0.48 and 0.73 µg g^−1^, respectively. As Liu and others (2019) have shown earlier, increased thallium content in bottom sediments and then in plants may be related to the influence of the steel industry and the steel-making area, which is the catchment area of the Kłodnica River.

The Kłodnica River is the main recipient of domestic and industrial wastewater of the Upper Silesian agglomeration. In terms of chromium content, the Kłodnica River shows its increased content in both water and bottom sediments. At the fourth sampling point, the chromium concentration in the bottom sediment was as much as 57.5 mg kg^−1^. Such strong contamination of bottom sediments with chromium caused an increase in this element’s concentration in coastal plants, in such a way, that in *Solidago virgaurea* L. the chromium concentration was 16.00 mg kg^−1^. Cr comes from anthropogenic pollution sources (Reis et al. 2019).

### Assessment of the Degree of Sediment Contamination

Arsenic has similar chemical and physical properties and geochemical behaviors to that of Sb and, hence, usually coexists with Sb in many environments. Traffic emission, coal combustion, Zn–Pb smelting and Sb mining and smelting are the most important Sb pollution sources (Bi et al. 2011). Although their presence in the environment has different environmental contexts, their increased amounts have usually been connected with human activity and are quite commonly used indicators of natural environment conditions (Wilson et al. 2010). In our research, a clear correlation was found between the concentration of arsenic and antimony in the bottom sediments of the Kłodnica River. The directly proportional relationship between the generally elevated and high concentrations of As and Sb in the sediments (the correlation coefficient was 0.90) indicates the anthropogenic origin of pollution. Sharifi et al. (2016) results show that the As(V) species is effectively immobilized by sorption or co-precipitation within the oxidized sediments, in agreement with the expected high affinity for mineral surfaces.

Values of the calculated indexes of pollutants are shown in Table 5. The I_geo_ values change in a wide range: from class 1 (uncontaminated to moderately polluted—for Cu and Zn) at the first sampling point to 5 (highly polluted to extremely polluted for Ba and Fe) at the K4 sampling point. The results of I_geo_ calculations showed that the highest values of this coefficient were found for the bottom sediments collected at point K4 located in Gliwice Łabędy. The Kłodnica River, flowing through the central part of the Upper Silesian Metropolis, is contaminated in many places, flowing through cities such as Katowice, Ruda Śląska and Gliwice. For this reason, at K4 point, the condition of the Kłodnica River reaches class 5 for Ba and Fe, which proves that the river is extremely polluted. At the K4 collection point, *I*_geo_ was also class 4, i.e., highly polluted for elements such as V, Cu and Zn. At this point, the third degree of pollution in the *I*_geo_ classification was also indicated for Cr and Sr.

**Table 5:**
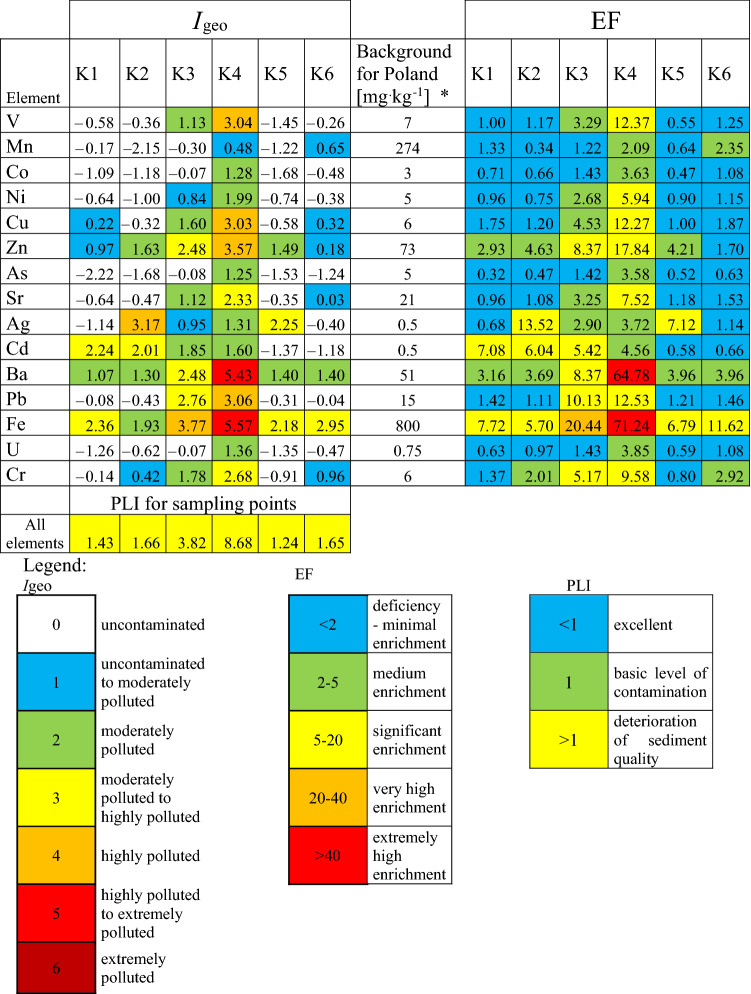
Values of the calculated indexes of pollutants; K1-K6 sampling points; EF (enrichment factor), *I*_geo_ (geoaccumulation index), PLI (pollution load index)

*Lis and Pasieczna (1998)

**Table 6:**
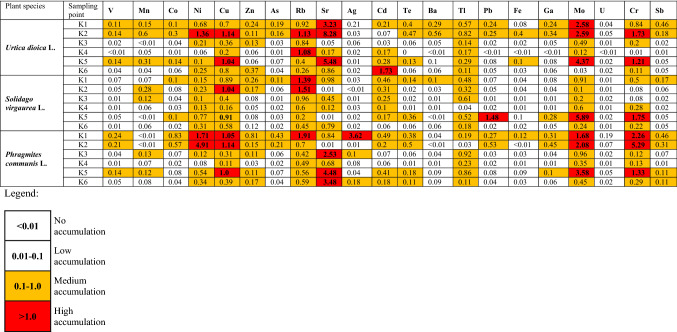
Estimation of bioaccumulation factor (BAF) of analyzed elements from plant species (*Urtica dioica* L., *Solidago virgaurea* L., *Phragmites communis* L.), K1-K6 sampling points

The obtained *I*_geo_ results clearly show an increase in the contamination with elements toward the runoff of the Kłodnica River. The quality of bottom sediments deteriorates from point K1 to point K4, then the condition of the river improves (river self-cleaning processes, flowing through areas subject to agricultural pressure and a decrease in pollutants flowing into the river). An average contamination to highly polluted bottom sediments at K5, i.e., Ujazd, has been shown for Ag and Fe, as well as being moderately polluted for Zn and Ba. The enrichment factor for Ba and Fe exceeds 30 (extremely high enrichment), and for Fe exceeded 20 (very high enrichment), and the remaining elements show significant or moderate enrichment of the soil.

The PLI index, as a more universal parameter, allows one to compare the studied sampling points regarding the degree of contamination. PLI was calculated taking into account sampling points separately for each element. Regardless of the calculation method, the PLI was usually > 1, indicating a deterioration in sediment quality. Although, *I*_geo_ showed moderate pollution and EF medium enrichment for arsenic, the PLI index for this element was under 1, which means that Kłodnica River sediment, in terms of all sampling points, is not contaminated by this element. Taking into account all tested elements for sampling points, the PLI indicates deterioration of sediment quality for all sampling points.

### Bioaccumulation of Elements in Plants

The bioaccumulation factor (BAF) is a coefficient that is used to measure the metal uptake of a plant. Values > 1 have been used to evaluate the potential of plant species for phytoextraction and phytostabilization of metals in the substrate. The BAF of the studied plant species was in the medium (0.1–1.0) and higher (> 1.0) range for almost all the analyzed metals (Table 6). The bioaccumulation factor in our study shows the ability of each plant species to accumulate potential toxic elements in their tissues at almost every sampling point. For the trace elements, BAF values were in the order Sr > Mo > Rb > Cu > Cr > Ni > Cd > Tl > Zn for nettle (*Urtica dioica* L.). For *Solidago virgaurea* L. the following order applies Mo > Rb > Cu > Cr > Sr > Tl > Ni > Cd > Zn. Regarding *Phragmites communis* L. the average BAF value was as follows Sr > Cr > Mo > Ni > Rb > Cu > Tl > Zn > Cd.

Among all elements, strontium had the highest bioaccumulation level in nettle and common reed plants. The highest value of 8.28 for the bioaccumulation factor of strontium was the nettles at the second sampling point.

For molybdenum, BAF values were obtained ranging from (4.37–0.12), with emphasis on *Urtica dioica* L. However, for this element and for other plants, the BAF value was high.

Nettle is a very suitable phytoextraction plant in highly polluted areas and is most efficient for the bioaccumulation for cadmium in potentially polluted areas (Balabanova et al. 2015). Among the examined plant species growing on the Kłodnica riverbank, nettle has a twice-higher BAF value for cadmium in comparison with goldenrod and common reed. This may be because that this element in the form of Cd^2+^ is a highly mobile metal and easily absorbed by plants to roots (Gitet et al. 2016).

Elevated levels of rubidium were noted in the Kłodnica River water. It is most likely related to the anthropogenic impact of the coal-burning industry (Jabłońska-Czapla et al. 2014a, b). The calculated BAF coefficient showed that this element is accumulated in the studied plant species at a similar level, in the range of 0.77–0.85.

The essential need of the plant tissue for Cu is 0.9 mg kg^−1^ (Balabanova et al. 2015). However, the determined content of the copper in our study was enriched to 14.5 mg kg^−1^ in the *Urtica dioica* L. at the fourth sampling point. For this element, BAF values were at medium to high levels at all sampling points for the three studied plant species.

Our research shows also increased thallium concentration in the bottom sediment and water of the Kłodnica River. BAF calculations showed that this element is largely bio-accumulated by plants. The average value for each plant was 0.37.

**Fig. 3:**
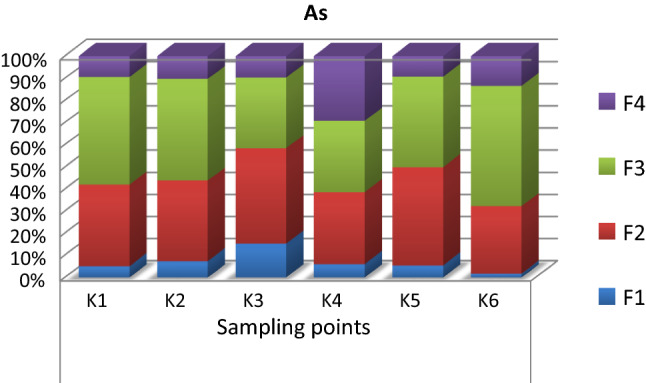
Sequential chemical extraction of the bottom sediments; F1 (mobile exchangeable fraction), F2 (mobile reducible fraction, associated with iron/manganese oxides), F3 (immobile oxidizable fraction, associated with organic substance and sulfides), F4 (immobile residual fraction, associated with non-silicate bound metals) and K1–K6 – the bottom sediment sampling points

Arsenic, especially its inorganic species, is one of the most toxic elements. At the K1 and K2 sampling points, the BAF value for this element was in the medium range. Arsenic in the bottom sediments was demobilized, which is confirmed by the results of sequential chemical extraction, indicating that As in the sediments occurred mainly in the immobile oxidizable fraction. Not surprisingly is the fact, that arsenic BAF value for studied plant species was quite low. The lowest arsenic BAF value was determined for plants collected at the fourth sampling point, where the bottom sediment contained the highest percentage of the most demobilized arsenic (Fig. 3).

## Conclusion

In the Kłodnica River basin, there are water reservoirs such as post-exploitation anthropogenic lakes: Dzierżno Duże, Pławniowice, and Dzierżno Małe. In the Kłodnica catchment area, there is the Gliwicki Channel, which connects the Oder River with the inland port in Gliwice. Waters in the Kłodnica River catchment area have always been of utility importance. They are important in the Śląskie Voivodeship in inland navigation or transport (Gliwice Canal). Waters in the Kłodnica River catchment also play an industrial role. The Pławniowice reservoir located in the Toszecki stream basin (inflow of the Kłodnica River) is used to supply water to the Gliwice smelter. Kłodnica River waters are also used in a small 75 kW hydroelectric power plant, which was built in the riverbed just before its mouth to the Dzierżno Duże reservoir.

A positive outcome is the currently reduced heavy metal pollution of the river waters. Their concentrations are now within the I and II classes of water purity (Regulation of Minister of Environment 2016). It is worth recalling that, in 1999, the Kłodnica River was an out-of-class river in this respect (below all ecological standards). The improvement of the situation in this matter is the effect of the ongoing restructuring of the economy in Upper Silesia. The liquidation of many obsolete and environmentally troublesome industrial plants has improved the condition of the Kłodnica River water.

The calculated environmental pollution factors such as PLI, *I*_geo_, EF and Sb/As factor confirmed that the Kłodnica River sediments are heavily polluted by heavy metals, especially in the first four sampling points, i.e., in the Upper Silesian agglomeration.

Our research has shown that the bottom sediments of the Kłodnica River are contaminated with metal(loid)s, including Pb, Zn, Ni and As, and among arsenic species, the As(V) form was quantitatively dominant; its highest concentration was 49.3 mg kg^−1^, with the organic species occurring extremely rarely. The highest concentration of arsenic, among the tested plants, occurred in *Phragmites communis* L. The concentrations of heavy metals in the bottom sediment of the Kłodnica River were extremely high and amounted to as much as 1302 mg kg^−1^ of zinc and 188 mg kg^−1^ of lead. As a result, coastal plants *Solidago virgaurea* L.*, Phragmites* L. and *Urtica dioica* L. were heavily contaminated by these elements.

The BAF of the studied plant species was in the medium (0.1–1.0) and higher (> 1.0) range for almost all the analyzed metals. Although the bottom sediments of the Kłodnica River were most heavily contaminated by lead and zinc, the calculations of the bioaccumulation factors of these metals in coastal plants were low. The highest bioaccumulation factors for the tested plant species were obtained for elements such as Sr, Mo, Rb, Cu, Cr and Ni.
